# A cluster of measles linked to an imported case, Finland, 2017

**DOI:** 10.2807/1560-7917.ES.2017.22.33.30595

**Published:** 2017-08-17

**Authors:** Elina Seppälä, Viktor Zöldi, Sakari Vuorinen, Satu Murtopuro, Ulpu Elonsalo, Janko van Beek, Anu Haveri, Mia Kontio, Carita Savolainen-Kopra, Taneli Puumalainen, Jussi Sane

**Affiliations:** 1 Department of Health Security, National Institute for Health and Welfare (THL), Helsinki, Finland; 2 European Programme for Intervention Epidemiology Training (EPIET), European Centre for Disease Prevention and Control (ECDC), Stockholm, Sweden; 3 Etelä-Savo Central Hospital District (Etelä-Savo Healthcare and Social Welfare District), Mikkeli Central Hospital, Mikkeli, Finland; 4 European Programme for Public Health Microbiology Training (EUPHEM), European Centre for Disease Prevention and Control, Stockholm, Sweden

**Keywords:** measles, outbreaks, vaccine-preventable diseases

## Abstract

One imported and five secondary cases of measles were detected in Finland between June and August 2017. The measles sequences available for five laboratory-confirmed cases were identical and belonged to serotype D8. The large number of potentially exposed Finnish and foreign individuals called for close cooperation of national and international public health authorities and other stakeholders. Raising awareness among healthcare providers and ensuring universally high vaccination coverage is crucial to prevent future clusters and outbreaks.

A young Italian adult was diagnosed with measles in Finland in June 2017. During the stay in Finland and subsequent travel to Estonia, the case exposed altogether several hundred persons to measles. As of 11 August, five secondary cases of measles have been detected. As the investigation is still ongoing, we present here the preliminary findings and implemented control measures regarding this cluster of measles.

## Case definition

In this investigation, a suspected case was any person who met clinical criteria (fever and maculopapular rash and cough/coryza/conjunctivitis). A probable case was any person who met clinical criteria and had an epidemiological link to a confirmed case. Confirmed cases were probable cases with laboratory evidence of infection with measles virus (detection of viral RNA with PCR and/or a positive IgM in serum).

## Description of the cluster

**Figure 1:**
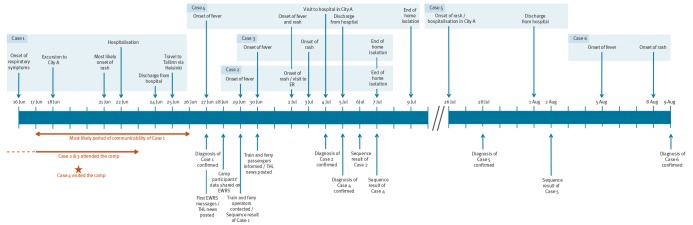
Timeline of the events, measles cases linked to importation, Finland, June–July 2017 (n = 6)

ER: emergency room; EWRS: Early Warning and Response System; THL: National Institute for Health and Welfare.

Index Case 1 was a young Italian adult who arrived in Finland on 11 June 2017 and attended an international camp in Finland from 12 to 25 June (Figure 1).

After developing fever and respiratory symptoms on 16 June and rash on 21 June, Case 1 was admitted to the hospital of City A and was isolated and monitored because enterovirus infection or measles was suspected. Serum and throat specimens were taken on 22 June. While the confirmatory laboratory results were still pending, the case was discharged and returned to the camp premises on 24 June. On 25 June, Case 1 travelled to Helsinki by train (without a designated seat, 3.5 hours) and to Tallinn by ferry (no cabin booked, 2.5 hours), staying one night before flying back to Italy. On 27 June, Case 1 was laboratory-confirmed for measles infection. The case self-reported to be vaccinated with two doses of measles-containing vaccine; however, the laboratory results contradicted this as the case tested negative for measles-specific IgG antibodies (Table).

**Table:** Summary of the laboratory findings measles cases linked to importation, Finland, June–July 2017 (n = 6)

	Vaccination status	Serology		qPCR	Genotype
		IgG	IgM		
**Case 1**	Unvaccinated				
Serum		Negative	Positive	ND	ND
Throat swab		ND	ND	Positive (Ct 25.76)	D8
**Case 2**	Unvaccinated				
Serum		Negative	Negative	ND	ND
Throat swab		ND	ND	Positive (Ct 23.61)	D8
**Case 4**	Unvaccinated				
Serum		Positive	Positive	ND	ND
Throat swab		ND	ND	Positive (Ct 23.77)	D8
**Case 5**	Unvaccinated				
Serum		Low positive	Positive	ND	ND
Throat swab		ND	ND	Positive (Ct 28.94)	D8
**Case 6**	2x MMR				
Serum		Positive	Negative	ND	ND
Throat swab		ND	ND	Positive (Ct 26.79)	D8

Ct: cycle threshold; ND: not done; MMR: measles-mumps-rubella vaccine; qPCR: real-time PCR; THL: National Institute for Health and Welfare.

Detection of measles-specific IgG and IgM antibodies was performed in serum samples (Euroimmun AG, Germany). Viral RNA was detected by real-time PCR directed against measles nucleoprotein gene. Infections with rubella virus and parvovirus B19 were ruled out. The sequences obtained for Cases 1, 2, 4, 5 and 6 are available via the MeaNS database (sequence ID: 114109, 114110, 114456, 116427 and 113656) and GenBank (accession numbers: MF409015, MF443206, and MF448447; accession numbers for Cases 5 and 6 not yet available).

**Figure 2:**
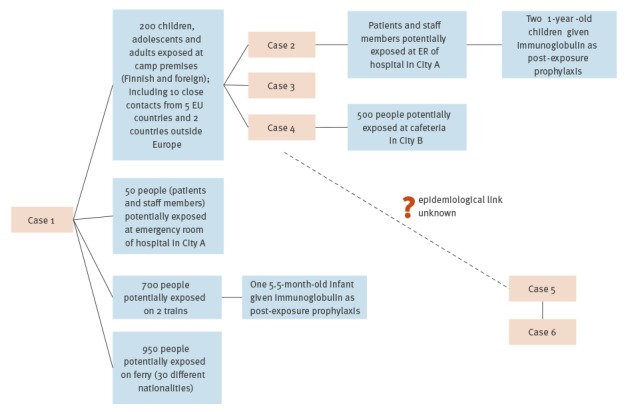
Epidemiological links between confirmed measles cases and potentially exposed groups, Finland, June–July 2017 (n = 6)

ER: emergency room; EU: European Union.

Subsequently, five secondary cases of measles were identified (Figures 1 and 2). Four of them were laboratory-confirmed (Cases 2, 4, 5 and 6). Case 3 was classified as a probable case (no laboratory testing because of parental objection). Cases 2 and 3 were adolescent siblings living in City A. Both attended a summer camp organised at the same premises as the international camp. Both were placed in home isolation. Case 4 was a person working in a cafeteria in City B who had visited the camp premises on 19 June. Cases 2, 3 and 4 had lunch in the same canteen used by other camp attendees including Case 1. Epidemiological investigations for Case 5 are still ongoing but so far no link between other cases has been established. It is possible that there is an unknown case in the transmission chain. Case 6 was a close contact of Case 5, identified during contact tracing. The case had been vaccinated with two doses of the measles-mumps-rubella (MMR) vaccine in childhood; all other secondary cases were unvaccinated (Table).

## Laboratory findings

Healthcare providers were asked to send all clinical samples from suspected measles cases to the measles reference laboratory of the Finnish National Institute for Health and Welfare (THL). Measles sequences available for five cases were identical and genotyped as D8 (Table). Based on the Measles Nucleotide Surveillance database, this strain was identical to strains isolated in Italy during 2017 and to a strain identified in a British traveller, and it shared very high nucleotide similarity (> 99.78%) with more than 600 sequences detected on five continents between 2015 and 2017, indicating recent global spread of nearly identical measles viruses [1,2].

Six suspected cases were discarded after laboratory analysis. In addition, three potentially exposed, asymptomatic people tested negative for measles.

### Control measures

Following the laboratory confirmation of Case 1 on 27 June, local, regional and national public health authorities promptly undertook contact tracing for the index and all subsequently identified cases. The contacts of suspected cases were mapped already while waiting for confirmation. Potentially exposed people were contacted after each case had been confirmed. THL communicated all available details of potentially exposed individuals of foreign nationality to the respective countries of origin. Countries in the European Union/European Economic Area (EU/EEA) were contacted through the Early Warning and Response System (EWRS), and non-EU/EEA countries through the World Health Organization (WHO) International Health Regulations (IHR) National Focal Points.

Hundreds of people were estimated to be potentially exposed to the confirmed cases (Figure 2). However, the degree and likelihood of exposure differed. Potentially exposed people were instructed to check their measles immunity status and to get vaccinated if necessary. They were also informed about the actions required if they experienced symptoms compatible with measles. Whenever possible, local health authorities contacted potentially exposed individuals personally. Other means of communicating this information included the publication of bulletins, both locally and by THL. In addition, all passengers on the involved trains and ferry for whom contact information (email address) was available were contacted through the respective operators in several languages. Healthcare providers were informed about the measles cases and given instructions on the actions required (prompt sampling, specimen shipment and isolation) if measles was suspected. The media also followed the event actively.

## Discussion

Measles outbreaks continue to occur in several EU/EEA countries, affecting especially countries where the vaccination coverage with two doses of the MMR vaccine is below the 95–99% threshold [3-6]. This cluster highlights the risk of importation of the measles virus by travellers originating from countries with intense transmission of the virus (a widespread outbreak is ongoing in Italy [3]), to countries without autochthonous transmission where the disease may spread among unprotected citizens.

In Finland, two doses of the MMR vaccine have been administered at 14–18 months and six years of age since the introduction of the vaccine in 1982 [7]. The national coverage has remained above 95% from the mid-1990s [8,9]. Since 1996, autochthonous measles transmission has stopped, and all transmission chains have been traced back to index cases who contracted the disease abroad [10]. However, in 2017, the coverage for birth cohort 2014 with at least one dose of MMR vaccine remains under 95% in one third of all Finnish health centre areas [11].

Cases 2, 3 and 4 were individuals belonging to the same immigrant community residing in Finland. The vaccination status of immigrant groups other than asylum seekers and refugees is currently not systematically screened, increasing the risk for the spread of measles and other vaccine-preventable diseases. Currently, some municipalities organise an introductory/screening visit to their healthcare centre when an immigrant receives rights to use the municipal healthcare services, but many do not make use of this opportunity. While children are followed up at well baby centres and schools, and some of the adults in occupational healthcare, there are other groups of immigrants who may live in Finland for years without any contact to healthcare services. The screening visits offered to immigrants should be promoted and used to check and update vaccination status.

In the absence of autochthonous transmission, recognition and control of measles may pose a challenge to healthcare professionals and public health authorities. Even in a highly immunised population, pockets of susceptible individuals may exist. The possibility of measles must be considered when encountering patients with compatible symptoms, especially travellers from a country or region known to have measles outbreaks. As Case 1 claimed to have been twice vaccinated, measles was not considered as the most likely cause of symptoms, and the case was discharged while the laboratory results were pending. However, the index case increased the awareness of physicians, and the subsequent cases were recognised promptly. Thanks to increased awareness, three asymptomatic, potentially exposed people were also tested for measles.

During this event, diagnostic tests for Cases 1 and 2 were delayed because of several reasons (public holidays, misunderstandings about specimen shipment). With highly contagious diseases such as measles, early detection and laboratory diagnostics as well as isolation of patients during the infectious period are essential to prevent further disease transmission. National guidelines for investigation and control of measles in Finland exist, and awareness of the guidelines among the healthcare professionals needs strengthening. This relatively rare event in Finland underlined the importance of having reference laboratory functions and epidemiological expertise integrated at the public health institute, facilitating efficient response to similar public health threats. Maintaining the integration was one priority recommendation in the recent Joint External Evaluation of Finland’s IHR core capacities [12].

This cluster also emphasised the importance of national and international cooperation of public health authorities, transportation operators and the media. Contact tracing in mass gatherings and public transportation may prove challenging, especially when individuals representing various nationalities have been present. When reaching out to individuals personally is not feasible, the role of timely and accurate media coverage as well as the assistance of transportation operators and their information channels become significant. In this instance, the train operator sent a message to all the 398 customers with registered email address travelling on the same train as Case 1, and 274 of them opened it. A somewhat lower opening rate (58%) was observed among ferry passengers (data only available for Finnish passengers).

## Conclusion

In Finland, a country with nationally high MMR vaccination coverage, extensive outbreaks of measles are unlikely to occur. However, transmission chains among unimmunised individuals linked to an imported case are possible. A prompt response and the cooperation of health authorities, the media and possible other stakeholders are crucial to interrupt transmission chains as soon as possible. Ensuring universally high vaccination coverage is essential to prevent clusters and outbreaks in the future.

## References

[1] Rota PA, Brown K, Mankertz A, Santibanez S, Shulga S, Muller CP, et al. Global distribution of measles genotypes and measles molecular epidemiology. J Infect Dis. 2011;204(Suppl 1):S514-23. 10.1093/infdis/jir118 21666208

[2] World Health Organization (WHO). Measles Nucleotide Surveillance (MeaNS) Database. Geneva: WHO. [Accessed 11 August 2017]. Available from: http://www.who-measles.org/Public/Web_Front/main.php

[3] European Centre for Disease Prevention and Control (ECDC). Ongoing outbreak of measles in Romania, risk of spread and epidemiological situation in EU/EEA countries (3 March 2017). Stockholm: ECDC; 2017. Available from: https://ecdc.europa.eu/sites/portal/files/media/en/publications/Publications/27-02-2017-RRA-Measles-Romania%2C%20European%20Union%20countries.pdf

[4] Moss WJ. Measles. Lancet. 2017;S0140-6736(17)31463-0. (Forthcoming). 10.1016/S0140-6736(17)31463-0 28673424

[5] Plans-Rubió P Evaluation of the establishment of herd immunity in the population by means of serological surveys and vaccination coverage. Hum Vaccin Immunother. 2012;8(2):184-8. 10.4161/hv.18444 22426372

[6] Plans-Rubió P Why does measles persist in Europe? Eur J Clin Microbiol Infect Dis. 2017; (Forthcoming). 10.1007/s10096-017-3011-y 28550369

[7] Rose A Measles eliminated in Finland since 1996 – will it last? Euro Surveill. 2003;7(3):2150.

[8] Davidkin I Kontio M Paunio M Peltola H MMR vaccination and disease elimination: the Finnish experience. Expert Rev Vaccines. 2010;9(9):1045-53. 10.1586/erv.10.99 20822347

[9] Paunio M Virtanen M Peltola H Cantell K Paunio P Valle M Increase of vaccination coverage by mass media and individual approach: intensified measles, mumps, and rubella prevention program in Finland. Am J Epidemiol. 1991;133(11):1152-60. 10.1093/oxfordjournals.aje.a115827 2035518

[10] Kantele A Valtonen K Davidkin I Martelius T Võželevskaja N Skogberg K Travellers returning with measles from Thailand to Finland, April 2012: infection control measures. Euro Surveill. 2012;17(22):20184. 22687913 10.2807/ese.17.22.20184-en

[11] National Institute for Welfare and Health (TFL). Vaccination coverage good among young children in Finland, but measles epidemics possible. Helsinki: THL; Feb 2017. Available from: https://www.thl.fi/fi/web/thlfi-en/-/while-vaccination-coverage-among-children-in-finland-is-good-measles-epidemics-are-possible

[12] World Health Organization (WHO). Joint external evaluation of IHR core capacities of the Republic of Finland. Mission report. Geneva: WHO; March 2017. Licence: CC BY-NC-SA 3.0 IGO. Available from: https://extranet.who.int/spp/sites/default/files/jeeta/WHO-WHE-CPI-2017.24-Report-eng.pdf

